# Standard errors of non-standardised and age-standardised relative survival of cancer patients

**DOI:** 10.1038/bjc.2011.560

**Published:** 2011-12-15

**Authors:** L Jansen, T Hakulinen, H Brenner

**Affiliations:** 1 Division of Clinical Epidemiology and Aging Research, German Cancer Research Center (DKFZ), POB 10 19 49, Heidelberg D-69009, Germany; 2 Finnish Cancer Registry, Helsinki, Finland

**Keywords:** epidemiologic methods, registries, survival

## Abstract

**Background:**

Relative survival estimates cancer survival in the absence of other causes of death. Previous work has shown that standard errors of non-standardised relative survival may be substantially overestimated by the conventionally used method. However, evidence was restricted to non-standardised relative survival estimates using Hakulinen's method. Here, we provide a more comprehensive evaluation of the accuracy of standard errors including age-standardised survival and estimation by the Ederer II method.

**Methods:**

Five- and ten-year non-standardised and age-standardised relative survival was estimated for patients diagnosed with 25 common forms of cancer in Finland in 1989–1993, using data from the nationwide Finnish Cancer Registry. Standard errors of mutually comparable non-standardised and age-standardised relative survival were computed by the conventionally used method and compared with bootstrap standard errors.

**Results:**

When using Hakulinen's method, standard errors of non-standardised relative survival were overestimated by up to 28%. In contrast, standard errors of age-standardised relative survival were accurately estimated. When using the Ederer II method, deviations of the standard errors of non-standardised and age-standardised relative survival were generally small to negligible.

**Conclusion:**

In most cases, overestimations of standard errors are effectively overcome by age standardisation and by using Ederer II rather than Hakulinen's method.

Relative survival reflects the survival cancer patients would be expected to have in the absence of competing causes of death and is commonly reported by cancer registries. It is computed as the ratio between absolute survival of cancer patients and expected survival in the absence of cancer. The latter is estimated from life tables of the general population (Ederer *et al*, 1961), mostly by the so-called Ederer II method (Ederer and Heise, 1959) or Hakulinen's method (Hakulinen, 1982).

The variance of relative survival is a function of the absolute and expected survival as well as the variance of absolute survival and expected survival and the covariance between absolute and expected survival (see Appendix). In the computation of the standard error of relative survival it is commonly assumed that the variance of expected survival is zero (which implies that the covariance with absolute survival is also zero), as the database underlying the population life tables is usually very large. Therefore, the standard error is commonly computed as the ratio between the standard error of absolute survival and the expected survival (Parkin and Hakulinen, 1991; Estéve *et al*, 1994). If the assumption of zero variance of expected survival does not hold, however (for reasons discussed below), then the variance of relative survival may be over- or underestimated, depending, among other factors, on the relative magnitude of the variance of expected survival and the covariance of absolute and expected survival (see Appendix ).

Brenner and Hakulinen (2005) empirically assessed the validity of the commonly used standard error definition (ignoring variance of expected survival) using data from the nationwide Finnish Cancer Registry. They computed conventional and bootstrap standard errors for 5- and 10-year non-standardised (crude) absolute, expected and relative survival for patients with 25 common forms of cancer in Finland in 1989. Expected survival was computed according to Hakulinen's method. When applying the conventional standard error definition, standard errors were overestimated by up to 17% for 5-year and 32% for 10-year non-standardised relative survival. This overestimation can be explained by non-zero standard errors for expected survival that may arise, as the expected survival is still subject to random variation due to random variation of the age distribution of the sample. Furthermore, absolute and expected survival may often be positively correlated, as survival typically decreases with increasing age in cancer patients as well as in the general population. To overcome the overestimation of standard errors, Brenner and Hakulinen (2005) suggested to compute bootstrap standard errors for relative survival and provided extensions of publicly available *SAS* macros for survival analysis (*period* and *periodh*; Brenner *et al*, 2002) that allow computing these standard errors.

In addition to non-standardised relative survival, age-standardised relative survival is commonly reported to compare survival across populations with different age structures. Age-standardised relative survival is commonly computed as the weighted sum of age-specific relative survival, using weights according to standard age distributions of cancer patients such as the International Cancer Survival Standard (ICSS; Verdecchia *et al*, 1999; Corazziari *et al*, 2004). Standard errors of these survival estimates are defined as the square root of the sum of the squared weighted age-stratified standard errors of relative survival. Thus, the same assumptions as in the computation of the standard error of non-standardised relative survival apply, but now on the level of the age-stratified survival estimates. As the age variability within these age groups is much smaller, the correlation of absolute and expected survival and the standard error of expected survival may be much lower. Thus, the conventional method to estimate the standard error of age-standardised relative survival might result in more accurate estimates.

To our knowledge, however, no previous study has assessed the validity of the conventional method in the context of age-standardised survival. This is particularly important, as age-standardised estimates are frequently reported. In addition, the validity of the method in the context of the Ederer II method, which is now increasingly applied, has not been investigated yet. Thus, the aim of this paper was to assess the validity of the conventional method to estimate standard errors of age-standardised relative survival and to estimate the accuracy of the conventional method when expected survival is computed according to either the Ederer II method or Hakulinen's method. Bootstrap standard errors of non-standardised and age-standardised relative survival were compared with standard errors computed according to the standard procedure. In addition, the random error of non-standardised expected survival and the correlation between non-standardised expected and absolute survival were estimated to check the assumptions of negligible variation of expected survival and negligible correlation.

## Materials and methods

The analysis was based on data from the population-based Finnish Cancer Registry, which has been operating for more than 50 years, covers the whole population of Finland (about 5.4 million people), and is well known for its data quality and completeness (Teppo *et al*, 1994). Patients aged 15 years or older with a first diagnosis of one of 25 common forms of cancer between 1989 and 1993 were included. These years of diagnosis were selected to ensure comparability with results of our earlier work on non-standardised relative survival, which had pertained to patients diagnosed in 1989, the most recent cohort of patients for whom 10-year survival had been completed at the time of our previous analysis. To avoid problems due to sparse data due to age stratification, the current analysis was extended to 5 years of diagnosis, that is, 1989–1993. Reproducibility of the results was assessed by repeating all analyses separately for patients diagnosed between 1979 and 1983 and between 1984 and 1988. As the patterns regarding the standard errors for non-standardised and age-standardised survival were generally very similar, results for these groups of patients are not shown separately.

For each cancer site, 5- and 10-year non-standardised absolute, expected and relative survival and age-standardised relative survival and the corresponding standard errors in % units were computed. Expected survival was calculated based on age-, sex- and calendar period-specific population life tables of Finland using both Hakulinen's (Hakulinen, 1982) and the Ederer II method (Ederer and Heise, 1959). Because available life tables of Finland were limited to ages up to 95 years, expected survival for cancer patients older than 95 years were based on the survival estimate of the general population of age 95 years. Age-standardised survival was computed as weighted average of age-specific survival, defining the age groups (15–44, 45–54, 55–64, 65–74, 75+ years) according to the ICSS (Corazziari *et al*, 2004) and the weights for each site and gender proportional to the numbers of patients at diagnosis in these age groups. The definition of weights was done in order to make the age-standardised and non-standardised survival figures mutually comparable.

For the calculation of the standard error of non-standardised and age-standardised relative survival, two methods were employed. The first method followed common practice in the analysis of population-based cancer registry data: Standard errors of relative survival were obtained by dividing the standard errors of absolute survival, which were computed according to Greenwood's method (Greenwood, 1926), by the expected survival (Ederer *et al*, 1961). For age-standardised survival, the ratio is computed between age-stratified estimates. The standard error of the age-standardised relative survival is estimated by the square root of the sum of the squared weighted age-stratified standard errors of relative survival. In the second method, the standard errors of relative survival were estimated by non-parametric bootstrap analysis as the standard deviation of the respective point estimate in 10 000 bootstrap samples. Each bootstrap sample was obtained by sampling with replacement from the patient population for a given cancer in the cancer registry, the same number of patients as in the original data set. Conventional and bootstrap standard errors were compared by computing ratios between the estimates.

To investigate the assumption of negligible standard error of expected survival and negligible correlation between absolute and expected survival, standard errors for non-standardised expected survival were estimated by bootstrapping, and Pearson's correlation coefficients of the point estimates of non-standardised absolute and expected survival in the bootstrap samples were calculated.

## Results

Table 1 shows the numbers of patients and their age distribution for each cancer site. Breast cancer was the most commonly diagnosed cancer in 1989–1993 (*N*=13 278), followed by lung (*N*=10 127), colorectal (*N*=8620) and prostate cancer (*N*=7753). Median age at diagnosis ranged from 34 years for testicular cancer to 74 years for prostate cancer.

Results for Hakulinen's method are shown in Table 2 for 5-year survival, and in Table 3 for 10-year survival. Five- and ten-year non-standardised absolute survival varied strongly by cancer site, ranging from 89.7 and 88.1% for testicular cancer to 1.8 and 1.1% for pancreatic cancer. Standard errors of 5- and 10-year absolute survival ranged from 0.25 and 2.84 to 0.18 and 2.98, respectively.

Non-standardised expected survival computed by Hakulinen's method was highest for testicular cancer, as these patients are on average younger than patients of the other cancer sites, and lowest for prostate cancer because of their relatively high age at diagnosis. Standard errors of 5- and 10-year non-standardised expected survival ranged from 0.13 and 0.22 for breast cancer to 0.93 and 1.53 for eye cancer. For all cancer sites, they were higher for 10-year than for 5-year survival and, although lower than standard error of absolute survival, generally not negligible.

Non-standardised absolute and expected survival was moderately positively correlated for both 5- and 10-year survival (ranges across cancer sites: 0.09–0.60 and 0.09–0.59, respectively).

Comparing standard errors of relative survival computed by the conventional and bootstrap method, conventional standard errors of 5-year non-standardised relative survival were overestimated by more than 5% for eight cancer sites and by more than 10% for Hodgkin's lymphoma (11%) and thyroid gland cancer (17%). Median overestimation across the 25 cancer sites was 4%. For 10-year non-standardised relative survival, overestimation was mostly larger (median: 6%). For seven cancer sites, it was larger than 10%. The highest overestimation was observed for thyroid gland cancer (26%). For age-standardised relative survival, deviations between the conventional and bootstrap standard errors were negligible (within ±1% for 16 of 25 cancer sites for 5-year survival and for 15 cancer sites for 10-year survival). Strongest overestimations were found for laryngeal cancer for 5-year (3%) and cancer of the breast, urinary bladder and corpus uteri for 10-year (3%) age-standardised relative survival.

The analysis was repeated with the computation of expected survival according to the Ederer II method (Tables 4 and 5). Non-standardised expected survival computed by the Ederer II method was overall higher than the estimates computed by Hakulinen's method. Standard errors of 5- and 10-year non-standardised expected survival were comparable, ranging from 0.15 and 0.27 for breast cancer to 1.55 and 4.01 for gallbladder cancer, respectively.

Correlations between non-standardised absolute and expected survival, which were based on the Ederer II method, were generally lower than the correlations in the context of Hakulinen's method (ranges across cancer sites: 0.03–0.32 and 0.03–0.32, respectively).

Conventional standard errors of relative survival were generally smaller when computed by the Ederer II instead of the Hakulinen's method. Deviations between the conventional standard errors and the bootstrap standard errors of non-standardised relative survival were negligible for 5-year survival (median deviation: 2%) and small for 10-year survival (median deviation: 3%). Deviations were largest for cancer of the thyroid gland and corpus uteri for 5-year survival (4%), and for gallbladder and urinary bladder cancer (−6 and 6%) for 10-year survival. The accuracy of the standard errors of age-standardised relative survival was again higher with a median deviation of 0% for 5-year and 10-year survival, and a maximal deviation of 4 and 6%, respectively.

## Discussion

Our results confirm previous observations that conventional standard errors for non-standardised relative survival, which were computed according to Hakulinen's method, may often be substantially overestimated reaching percentage deviations of up to 28% in the examples assessed in this article. Overestimations were overall larger in case of long survival times, that is, for 10- than for 5-year survival, and in case of higher correlations between absolute and expected survival. Largest overestimations were observed for thyroid cancer and Hodgkin's lymphoma. Both cancer sites had the highest correlation between absolute and expected survival, which can be explained by the large age gradient in prognosis of patients with these two malignancies. We could show, however, that standard errors for age-standardised relative survival, which were computed by Hakulinen's method, were accurately estimated by the conventional standard error method. When using the Ederer II method, deviations of the standard errors of non-standardised relative survival were small to negligible, and standard errors of age-standardised survival were accurately estimated.

The commonly used definition of the standard error of relative survival is based on the assumption of zero standard error of expected survival. However, non-negligible standard errors for non-standardised expected survival were observed, which can be explained by the random variation of the age distribution of the sample. Furthermore, for many cancer sites, a substantial positive correlation between non-standardised absolute survival and expected survival computed by Hakulinen's method was observed, which can be explained by the common variation of absolute and expected survival, as both estimates decrease with increasing age. Within age groups used for age-standardisation random variation of the age distribution and, hence, variance of expected survival and covariance of absolute and expected survival is smaller.

Overestimation of the standard error of non-standardised relative survival is a consequence of random error in expected survival, along with positive covariance of absolute and expected survival (Brenner and Hakulinen, 2005). Substantial overestimations were observed when expected survival was computed by Hakulinen's method. The age-standardisation computed as weighted averages of age-specific estimates overcomes the problem of overestimated standard errors of relative survival. When expected survival was computed by the Ederer II method, errors in the estimation of the standard errors of non-standardised relative survival were small to negligible. The difference between these two methods might be explained in part by smaller covariance of absolute and expected survival for the Ederer II than for Hakulinen's method, which is reflected by the smaller correlation coefficients in our bootstrap analysis.

Relative survival, computed as the ratio of absolute and expected survival, is often interpreted as an estimate of net survival when cancer was the only cause of death. However, it has been shown that this interpretation is valid only when survival with respect to cancer and survival with respect to other diseases are independent (Pohar Perme *et al*, 2011). This condition is only rarely fulfilled, as both survival proportions are usually affected by common covariates such as age. Comparison of the accuracy of methods such as Ederer I, II and Hakulinen for the estimation of net survival have usually been restricted to investigations of the point estimate (Pokhrel and Hakulinen, 2008; Hakulinen *et al*, 2011). These investigations have suggested that without applying regression modelling, the gold standard to estimate net survival may be age-standardised relative survival with expected survival computed by the Ederer II method and weights proportional to the number of patients at the beginning of the follow-up (Pokhrel and Hakulinen, 2008). In general, the bias in the estimation of net survival is smaller when using the Ederer II method than when using the Ederer I or Hakulinen's method and increases for the Ederer I and Hakulinen's method over time (Hakulinen *et al*, 2011). Our results on the standard error of relative survival support the recommendation of the Ederer II method, as both standard errors of non-standardised and age-standardised relative survival estimates were accurately estimated by the conventional method.

Recently, a new method to estimate net survival was proposed by Pohar Perme *et al* (2011). This new method provides an unbiased estimate for net survival without any modelling. The corresponding variance estimate has already been tested for accuracy by comparison with bootstrap estimates. Results from the empirical and theoretical investigations of this method are very promising. However, the method requires the absence of censoring and it has to be shown how estimates change in case of censoring.

The accuracy of the standard error of relative survival was estimated by the ratio between the conventional and bootstrap standard error. To test whether the measurement of bias in relative terms depends on the sample size, we re-run the analysis for the two most common cancer sites (breast and lung cancer) and the two cancer sites with the largest bias (thyroid cancer and Hodgkin's lymphoma) using a variable proportion (1, 2, 3, 4, 5, 10, 15, 20, 25, 30, 40,  … , 90%) of the complete data set. No indication for the dependence of relative bias on the sample size was found (data not shown).

In summary, our results show that overestimations of the standard errors can be substantially reduced by computing age-standardised survival. In age-standardised survival, the correlation between absolute and expected survival is substantially reduced. For the Ederer II method, standard errors of non-standardised and age-standardised relative survival were accurately estimated. Thus, our results support the recommendation to use the Ederer II method to estimate relative survival ratios.

## Figures and Tables

**Table 1:** Numbers and age distribution of patients aged 15 or more years with a first diagnosis of common forms of cancer, Finland, 1989–1993

		**Percentiles of age distribution (years)**				
	**No.**	**10th**	**25th**	**50th**	**75th**	**90th**
Oral cavity	1913	47	58	68	77	83
Oesophagus	971	55	63	72	79	85
Stomach	4691	51	62	71	79	84
Colorectal	8620	52	62	71	78	84
Liver	1017	52	63	71	78	84
Pancreas	3291	55	64	72	79	84
Larynx	549	49	58	65	73	79
Lung	10127	55	62	68	75	80
Skin melanoma	2445	35	45	58	71	80
Breast	13278	43	49	60	72	80
Urinary bladder	3546	54	63	71	78	83
Cervix	668	37	47	65	76	82
Corpus uteri	2852	51	58	66	73	80
Ovaries	2252	44	54	64	73	80
Prostate	7753	62	68	74	79	84
Testis	329	22	26	34	43	56
Kidneys	3074	49	58	67	74	80
Eye	281	40	50	63	71	80
Brain/nervous system	1486	29	40	54	67	75
Thyroid gland	1385	29	39	50	65	76
Non-Hodgkin's lymphoma	3356	41	54	66	75	81
Multiple myeloma	1166	55	63	71	78	84
Leukaemia	1884	41	56	68	77	83
Biliary passage and gallbladder	769	59	65	73	80	85
Hodgkin's lymphoma	553	20	27	39	58	73

**Table 2:** Non-standardised absolute, expected and relative survival and age-standardised relative survival after 5 years of patients aged 15 or more years with a first diagnosis of common forms of cancer, Finland, 1989–1993^a^

	**Non-standardised 5-year survival**									**Age-standardised 5-year survival**			
	**Conventional analysis**					**Bootstrap analysis**				**Conventional analysis**		**Bootstrap analysis**	
	**Abs**	**Exp**	**Rel**	**SE_Abs_**	**SE_RG_**	**SE_Exp_**	**Corr_AbsExp_**	**SE_RB_**	**SE_RG_/SE_RB_**	**Rel**	**SE_RG_**	**SE_RB_**	**SE_RG_/SE_RB_**
Oral cavity	51.0	79.7	64.1	1.14	1.43	0.43	0.26	1.38	1.04	63.7	1.49	1.48	1.01
Oesophagus	5.5	75.5	7.2	0.73	0.97	0.63	0.14	0.96	1.00	6.7	0.91	0.91	1.00
Stomach	16.9	76.2	22.2	0.55	0.72	0.29	0.23	0.71	1.01	21.0	0.70	0.68	1.02
Colorectal	39.4	77.9	50.5	0.53	0.68	0.20	0.29	0.66	1.03	49.7	0.69	0.67	1.02
Liver	3.0	77.3	3.9	0.54	0.70	0.58	0.13	0.70	1.00	3.5	0.62	0.63	0.99
Pancreas	1.8	76.9	2.3	0.23	0.30	0.32	0.11	0.30	0.98	2.0	0.26	0.26	1.00
Larynx	49.9	81.7	61.1	2.13	2.61	0.66	0.26	2.51	1.04	60.8	2.69	2.62	1.03
Lung	7.2	79.3	9.1	0.25	0.32	0.16	0.15	0.32	1.01	8.6	0.31	0.31	1.00
Skin melanoma	71.0	87.4	81.3	0.92	1.05	0.32	0.38	0.98	1.07	80.5	1.09	1.08	1.01
Breast	72.2	89.2	80.9	0.39	0.44	0.13	0.35	0.41	1.05	80.2	0.45	0.45	1.01
Urinary bladder	51.9	75.6	68.7	0.84	1.11	0.31	0.38	1.04	1.07	66.9	1.12	1.10	1.02
Cervix	48.7	86.4	56.4	1.94	2.24	0.64	0.44	2.08	1.08	53.5	2.04	2.03	1.00
Corpus uteri	69.4	87.5	79.3	0.87	0.99	0.27	0.40	0.92	1.08	78.0	0.99	0.97	1.02
Ovaries	32.6	88.2	37.0	0.99	1.12	0.31	0.29	1.09	1.03	35.4	1.04	1.04	1.00
Prostate	43.7	68.6	63.7	0.56	0.82	0.20	0.24	0.79	1.04	64.1	0.86	0.86	1.01
Testis	89.7	96.8	92.6	1.67	1.73	0.36	0.40	1.62	1.07	92.3	1.74	1.77	0.98
Kidneys	44.4	83.0	53.4	0.90	1.08	0.28	0.28	1.05	1.03	52.4	1.08	1.07	1.01
Eye	65.1	86.7	75.1	2.84	3.28	0.93	0.32	3.10	1.06	74.5	3.36	3.36	1.00
Brain/nervous system	29.1	91.2	31.9	1.18	1.29	0.31	0.35	1.26	1.03	30.1	1.06	1.05	1.02
thyroid gland	80.9	92.5	87.5	1.05	1.14	0.34	0.60	0.98	1.17	85.3	1.03	1.02	1.01
Non-Hodgkin's lymphoma	42.1	83.7	50.3	0.85	1.02	0.29	0.37	0.97	1.05	47.8	0.96	0.94	1.02
Multiple myeloma	21.3	77.9	27.3	1.20	1.54	0.54	0.34	1.46	1.05	24.8	1.37	1.38	0.99
Leukaemia	32.2	80.7	39.8	1.07	1.33	0.43	0.34	1.27	1.05	37.7	1.27	1.26	1.01
Gallbladder	5.5	77.1	7.1	0.82	1.06	0.68	0.09	1.06	1.00	7.1	1.09	1.08	1.00
Hodgkin's lymphoma	74.1	93.7	79.1	1.86	1.99	0.49	0.51	1.79	1.11	76.9	1.75	1.72	1.02

Abbreviations: Abs=absolute survival; Exp=expected survival; Rel=relative survival; SE_Abs_=standard error of absolute survival; SE_RG_=standard error of relative survival according to Greenwood formula; Corr_AbsExp_=correlation of absolute and expected survival in 10 000 bootstrap replications; SE_Exp_=standard error of expected survival; SE_RB_=standard error of relative survival according to bootstrap analysis

Standard errors are reported in % units.

a Expected survival was computed according to Hakulinen's method.

**Table 3:** Non-standardised absolute, expected and relative survival and age-standardised relative survival after 10 years of patients aged 15 or more years with a first diagnosis of common forms of cancer, Finland, 1989–1993^a^

	**Non-standardised 10-year survival**									**Age-standardised 10-year survival**			
	**Conventional analysis**					**Bootstrap analysis**				**Conventional analysis**		**Bootstrap analysis**	
	**Abs**	**Exp**	**Rel**	**SE_Abs_**	**SE_RG_**	**SE_Exp_**	**Corr_AbsExp_**	**SE_RB_**	**SE_RG_/SE_RB_**	**Rel**	**SE_RG_**	**SE_RB_**	**SE_RG_/SE_RB_**
Oral cavity	35.2	62.6	56.3	1.10	1.75	0.65	0.27	1.63	1.07	54.7	2.01	1.99	1.01
Oesophagus	3.6	55.6	6.5	0.60	1.08	0.90	0.13	1.06	1.02	6.0	1.11	1.10	1.01
Stomach	12.0	56.9	21.1	0.47	0.83	0.42	0.21	0.81	1.03	19.1	0.88	0.86	1.02
Colorectal	27.5	59.0	46.6	0.48	0.82	0.30	0.27	0.76	1.07	45.3	0.93	0.91	1.02
Liver	1.8	57.7	3.1	0.42	0.72	0.86	0.12	0.72	1.00	2.2	0.52	0.52	0.99
Pancreas	1.1	57.1	2.0	0.18	0.32	0.48	0.12	0.32	1.00	1.5	0.26	0.26	1.01
Larynx	33.2	65.0	51.0	2.01	3.09	1.04	0.25	2.93	1.05	50.2	3.54	3.49	1.01
Lung	3.7	60.5	6.0	0.19	0.31	0.24	0.15	0.31	1.01	5.3	0.29	0.29	1.01
Skin melanoma	58.6	76.0	77.1	1.00	1.31	0.52	0.39	1.16	1.14	75.4	1.55	1.52	1.02
Breast	56.4	78.8	71.6	0.43	0.55	0.22	0.36	0.49	1.11	69.8	0.62	0.60	1.03
Urinary bladder	34.3	55.3	61.9	0.80	1.44	0.45	0.37	1.28	1.12	56.3	1.53	1.49	1.03
Cervix	40.2	73.6	54.6	1.90	2.58	1.07	0.43	2.30	1.12	48.2	2.44	2.43	1.00
Corpus uteri	57.5	74.5	77.2	0.92	1.24	0.44	0.39	1.09	1.14	74.7	1.38	1.34	1.03
Ovaries	25.3	76.2	33.2	0.91	1.20	0.52	0.28	1.15	1.05	29.9	1.12	1.12	1.00
Prostate	20.3	44.5	45.6	0.46	1.03	0.27	0.25	0.97	1.06	45.4	1.19	1.18	1.01
Testis	88.1	93.4	94.4	1.78	1.91	0.67	0.39	1.74	1.10	94.5	2.32	2.37	0.98
Kidneys	30.8	66.9	46.0	0.84	1.25	0.45	0.28	1.18	1.06	43.0	1.27	1.25	1.02
Eye	47.0	74.4	63.2	2.98	4.00	1.53	0.32	3.72	1.08	60.7	4.39	4.36	1.01
Brain/nervous system	21.0	82.1	25.5	1.06	1.29	0.55	0.34	1.25	1.03	22.9	1.11	1.09	1.02
Thyroid Gland	75.1	85.0	88.3	1.16	1.37	0.58	0.59	1.08	1.26	84.0	1.39	1.38	1.01
Non-Hodgkin's lymphoma	29.3	68.7	42.7	0.78	1.14	0.46	0.36	1.07	1.07	38.4	1.13	1.12	1.01
Multiple myeloma	8.2	58.9	13.8	0.80	1.36	0.80	0.33	1.31	1.04	10.4	1.06	1.07	0.99
Leukaemia	16.7	64.3	26.0	0.86	1.34	0.66	0.31	1.27	1.05	22.2	1.26	1.25	1.01
Gallbladder	3.5	57.3	6.1	0.66	1.16	1.03	0.09	1.16	1.00	6.4	1.38	1.38	1.00
Hodgkin's lymphoma	66.7	87.7	76.0	2.01	2.29	0.85	0.51	1.96	1.17	71.4	2.08	2.03	1.02

Abbreviations: Abs=absolute survival; Exp=expected survival; Rel=relative survival; SE_Abs_=standard error of absolute survival; SE_RG_=standard error of relative survival according to Greenwood formula; Corr_AbsExp_=correlation of absolute and expected survival in 10 000 bootstrap replications; SE_Exp_=standard error of expected survival; SE_RB_=standard error of relative survival according to bootstrap analysis.

Standard errors are reported in % units.

a Expected survival was computed according to Hakulinen's method.

**Table 4:** Non-standardised absolute, expected and relative survival and age-standardised relative survival after 5 years of patients aged 15 or more years with a first diagnosis of common forms of cancer, Finland, 1989–1993^a^

	**Non-standardised 5-year survival**									**Age-standardised 5-year survival**			
	**Conventional analysis**					**Bootstrap analysis**				**Conventional analysis**		**Bootstrap analysis**	
	**Abs**	**Exp**	**Rel**	**SE_Abs_**	**SE_RG_**	**SE_Exp_**	**Corr_AbsExp_**	**SE_RB_**	**SE_RG_/SE_RB_**	**Rel**	**SE_RG_**	**SE_RB_**	**SE_RG_/SE_RB_**
Oral cavity	51.0	79.5	64.2	1.14	1.44	0.53	0.21	1.42	1.01	63.9	1.50	1.48	1.01
Oesophagus	5.5	79.6	6.9	0.73	0.92	1.09	0.16	0.89	1.03	6.6	0.89	0.88	1.00
Stomach	16.9	80.1	21.1	0.55	0.68	0.42	0.15	0.68	1.00	20.7	0.68	0.67	1.02
Colorectal	39.4	79.5	49.5	0.53	0.66	0.24	0.16	0.66	1.00	49.3	0.68	0.67	1.02
Liver	3.0	81.1	3.8	0.54	0.67	1.77	0.15	0.66	1.00	3.5	0.62	0.64	0.97
Pancreas	1.8	83.7	2.1	0.23	0.27	0.92	0.19	0.27	1.02	2.0	0.26	0.26	1.00
Larynx	49.9	82.5	60.5	2.13	2.59	0.74	0.11	2.58	1.00	60.5	2.67	2.68	1.00
Lung	7.2	82.5	8.8	0.25	0.31	0.27	0.14	0.31	1.02	8.5	0.31	0.31	0.99
Skin melanoma	71.0	87.8	80.9	0.92	1.05	0.35	0.26	1.02	1.02	80.5	1.09	1.07	1.02
Breast	72.2	89.5	80.6	0.39	0.43	0.15	0.21	0.43	1.01	80.3	0.45	0.45	1.01
Urinary bladder	51.9	77.0	67.4	0.84	1.09	0.35	0.22	1.07	1.02	66.7	1.11	1.10	1.02
Cervix	48.7	89.7	54.3	1.94	2.16	0.62	0.30	2.09	1.03	53.1	2.01	2.00	1.01
Corpus uteri	69.4	88.5	78.4	0.87	0.98	0.28	0.21	0.94	1.04	77.8	0.98	0.98	1.00
Ovaries	32.6	91.6	35.6	0.99	1.08	0.31	0.11	1.07	1.01	35.2	1.03	1.04	0.99
Prostate	43.7	68.5	63.8	0.56	0.82	0.23	0.18	0.81	1.02	63.9	0.86	0.85	1.01
Testis	89.7	97.2	92.2	1.67	1.73	0.34	0.19	1.70	1.02	92.2	1.73	1.80	0.96
Kidneys	44.4	84.6	52.4	0.90	1.06	0.31	0.15	1.04	1.02	52.1	1.06	1.07	1.00
Eye	65.1	87.8	74.2	2.84	3.24	0.90	0.17	3.23	1.00	74.0	3.30	3.32	0.99
Brain/nervous system	29.1	95.5	30.5	1.18	1.23	0.26	0.19	1.23	1.01	30.1	1.06	1.06	1.00
Thyroid gland	80.9	94.3	85.8	1.05	1.12	0.30	0.32	1.07	1.04	85.0	1.01	1.01	1.00
Non-Hodgkin's lymphoma	42.1	87.4	48.1	0.85	0.97	0.29	0.17	0.98	1.00	47.4	0.94	0.94	0.99
Multiple myeloma	21.3	82.3	25.8	1.20	1.46	0.61	0.28	1.42	1.02	24.5	1.35	1.34	1.01
Leukaemia	32.2	84.8	38.0	1.07	1.27	0.47	0.18	1.24	1.02	37.3	1.24	1.24	1.01
Gallbladder	5.5	80.2	6.8	0.82	1.02	1.55	0.03	1.03	0.99	6.9	1.05	1.05	1.00
Hodgkin's lymphoma	74.1	95.7	77.5	1.86	1.95	0.39	0.26	1.91	1.02	76.8	1.74	1.77	0.98

Abbreviations: Abs=absolute survival; Exp=expected survival; Rel=relative survival; SE_Abs_=standard error of absolute survival; SE_RG_=standard error of relative survival according to Greenwood formula; Corr_AbsExp_=correlation of absolute and expected survival in 10 000 bootstrap replications; SE_Exp_=standard error of expected survival; SE_RB_=standard error of relative survival according to bootstrap analysis.

Standard errors are reported in % units.

a Expected survival was computed according to the Ederer II method.

**Table 5:** Non-standardised absolute, expected and relative survival and age-standardised relative survival after 10 years of patients aged 15 or more years with a first diagnosis of common forms of cancer, Finland, 1989–1993^a^

	**Non-standardised 10-year survival**									**Age-standardised 10-year survival**			
	**Conventional analysis**					**Bootstrap analysis**				**Conventional analysis**		**Bootstrap analysis**	
	**Abs**	**Exp**	**Rel**	**SE_Abs_**	**SE_RG_**	**SE_Exp_**	**Corr_AbsExp_**	**SE_RB_**	**SE_RG_/SE_RB_**	**Rel**	**SE_RG_**	**SE_RB_**	**SE_RG_/SE_RB_**
Oral cavity	35.2	62.4	56.5	1.10	1.75	0.88	0.31	1.68	1.04	55.0	2.04	2.02	1.01
Oesophagus	3.6	63.8	5.6	0.60	0.94	2.39	0.01	0.95	0.98	5.6	1.00	0.99	1.00
Stomach	12.0	64.0	18.7	0.47	0.74	0.85	0.20	0.73	1.01	18.5	0.83	0.82	1.01
Colorectal	27.5	61.4	44.8	0.48	0.78	0.46	0.25	0.77	1.02	44.8	0.91	0.91	1.00
Liver	1.8	70.7	2.5	0.42	0.58	3.05	0.25	0.57	1.03	2.2	0.52	0.52	0.99
Pancreas	1.1	73.7	1.5	0.18	0.25	2.42	0.16	0.25	1.01	1.4	0.25	0.25	0.98
Larynx	33.2	66.9	49.6	2.01	3.00	1.36	0.22	2.91	1.03	49.1	3.39	3.40	1.00
Lung	3.7	68.0	5.4	0.19	0.27	0.63	0.16	0.27	1.02	5.1	0.28	0.27	1.01
Skin melanoma	58.6	77.0	76.0	1.00	1.29	0.62	0.29	1.27	1.02	75.2	1.54	1.53	1.01
Breast	56.4	79.7	70.8	0.43	0.54	0.27	0.28	0.52	1.05	69.7	0.62	0.61	1.02
Urinary bladder	34.3	58.5	58.6	0.80	1.36	0.58	0.32	1.28	1.06	55.9	1.50	1.47	1.02
Cervix	40.2	81.7	49.2	1.90	2.32	1.16	0.28	2.23	1.04	47.4	2.29	2.30	1.00
Corpus uteri	57.5	76.9	74.8	0.92	1.20	0.53	0.26	1.17	1.03	74.2	1.35	1.33	1.02
Ovaries	25.3	83.7	30.2	0.91	1.09	0.68	0.17	1.08	1.01	29.5	1.08	1.10	0.99
Prostate	20.3	44.3	45.7	0.46	1.03	0.35	0.25	1.00	1.03	44.8	1.17	1.14	1.02
Testis	88.1	94.1	93.6	1.78	1.90	0.70	0.17	1.90	1.00	94.1	2.29	2.44	0.94
Kidneys	30.8	70.2	43.9	0.84	1.19	0.62	0.26	1.14	1.04	42.5	1.25	1.24	1.01
Eye	47.0	76.7	61.3	2.98	3.88	1.67	0.27	3.75	1.04	59.6	4.13	4.16	0.99
Brain/nervous system	21.0	92.1	22.7	1.06	1.15	0.57	0.08	1.15	1.00	22.6	1.08	1.08	1.00
Thyroid gland	75.1	88.9	84.5	1.16	1.31	0.56	0.28	1.27	1.03	83.5	1.34	1.37	0.98
Non-Hodgkin's lymphoma	29.3	76.4	38.4	0.78	1.03	0.60	0.19	1.02	1.01	37.6	1.08	1.08	1.00
Multiple myeloma	8.2	71.1	11.5	0.80	1.13	1.25	0.24	1.10	1.02	10.2	1.01	1.03	0.98
Leukaemia	16.7	72.9	22.9	0.86	1.18	0.87	0.30	1.13	1.05	21.4	1.16	1.14	1.02
Gallbladder	3.5	61.3	5.7	0.66	1.08	4.01	0.03	1.15	0.94	6.0	1.26	1.33	0.94
Hodgkin's lymphoma	66.7	92.5	72.1	2.01	2.17	0.62	0.30	2.11	1.03	70.9	1.97	1.95	1.01

Abbreviations: Abs=absolute survival; Exp=expected survival; Rel=relative survival; SE_Abs_=standard error of absolute survival; SE_RG_=standard error of relative survival according to Greenwood formula; Corr_AbsExp_=correlation of absolute and expected survival in 10 000 bootstrap replications; SE_Exp_=standard error of expected survival; SE_RB_=standard error of relative survival according to bootstrap analysis.

Standard errors are reported in % units.

a Expected survival was computed according to the Ederer II method.

## APPENDIX

### Some mathematical details

Applying the delta method, the variance of relative survival (var (*R*(*t*)) is a function of absolute (*A*(*t*)) and expected survival (*E*(*t*)) as well as the variance of absolute survival (var(*A*(*t*)) and expected survival (var(*E*(*t*)) and the covariance between absolute and expected survival cov(*A*(*t*), *E*(*t*)) (Brenner and Hakulinen, 2005):

It is commonly assumed that the variance of expected survival and the covariance between absolute and expected survival are zero. Therefore, the variance of relative survival is conventionally computed using the Greenwood formula by:

The bias introduced by using the Greenwood formula is given by:

The relative bias is given by
